# Characteristics and related factors of emergency department visits, readmission, and hospital transfers of inpatients under a DRG-based payment system: A nationwide cohort study

**DOI:** 10.1371/journal.pone.0243373

**Published:** 2020-12-09

**Authors:** Pei-Fang Huang, Pei-Tseng Kung, Wen-Yu Chou, Wen-Chen Tsai

**Affiliations:** 1 Department of Health Services Administration, China Medical University, Taichung, Taiwan, R.O.C; 2 Department of Superintendent, Taichung Tzu Chi Hospital, Buddhist Tzu Chi Medical Foundation, Taichung, Taiwan, R.O.C; 3 Department of Healthcare Administration, Asia University, Taichung, Taiwan, R.O.C; 4 Department of Medical Research, China Medical University Hospital, China Medical University, Taichung, Taiwan, R.O.C; Istituto Di Ricerche Farmacologiche Mario Negri, ITALY

## Abstract

**Objectives:**

Taiwan has implemented the Diagnosis Related Groups (DRGs) since 2010, and the quality of care under the DRG-Based Payment System is concerned. This study aimed to examine the characteristics, related factors, and time distribution of emergency department (ED) visits, readmission, and hospital transfers of inpatients under the DRG-Based Payment System for each Major Diagnostic Category (MDC).

**Methods:**

We conducted a retrospective cohort study using data from the National Health Insurance Research Database (NHIRD) from 2012 to 2013 in Taiwan. Multilevel logistic regression analysis was used to examine the factors related to ED visits, readmissions, and hospital transfers of patients under the DRG-Based Payment System.

**Results:**

In this study, 103,779 inpatients were under the DRG-Based Payment System. Among these inpatients, 4.66% visited the ED within 14 days after their discharge. The factors associated with the increased risk of ED visits within 14 days included age, lower monthly salary, urbanization of residence area, comorbidity index, MDCs, and hospital ownership (p < 0.05). In terms of MDCs, Diseases and Disorders of the Kidney and Urinary Tract (MDC11) conferred the highest risk of ED visits within 14 days (OR = 4.95, 95% CI: 2.69–9.10). Of the inpatients, 6.97% were readmitted within 30 days. The factors associated with the increased risk of readmission included gender, age, lower monthly salary, comorbidity index, MDCs, and hospital ownership (p < 0.05). In terms of MDCs, the inpatients with Pregnancy, Childbirth and the Puerperium (MDC14) had the highest risk of readmission within 30 days (OR = 20.43, 95% CI: 13.32–31.34). Among the inpatients readmitted within 30 days, 75.05% of them were readmitted within 14 days. Only 0.16% of the inpatients were transferred to other hospitals.

**Conclusion:**

The study shows a significant correlation between Major Diagnostic Categories in surgery and ED visits, readmission, and hospital transfers. The results suggested that the main reasons for the high risk may need further investigation for MDCs in ED visits, readmissions, and hospital transfers.

## 1. Introduction

Many countries have successfully implemented Prospective Payment Systems (PPSs) to increase hospitals' efficiency in treating patients. The Diagnosis Related Groups (DRGs) belong to the PPS. In 1983, this system was officially adopted by the US Medicare [1], with many countries following suit [2].

After implementing DRG-based payment systems in the USA, Germany, Korea, and other countries, the average length of hospitalization was significantly reduced [3–6]. Some studies have analyzed this based on specific or common surgical cases, and the results showed that healthcare quality either improved or showed no significant change after system implementation [7–9]. In addition to the common measure of "length of hospitalization," healthcare quality indicators also include the post-discharge readmission rate (such as ED visits and readmission rate), hospital transfer rate, and intensity of care (i.e., the number of orders during one’s hospitalization) [7–9]. Although DRG-based payment systems are widely used, they still have some hidden problems. Studies suggest that hospitals may discharge patients earlier due to cost considerations, which could adversely impact healthcare quality [10]. Many studies showed that there is a reduction in healthcare quality, such as earlier hospital discharge, increased readmission, and unsatisfactory patient care [11–15].

Since 2010, Taiwan has implemented the National Health Insurance Taiwanese Diagnosis Related Groups (Tw-DRG) payment system in stages. In this system, there is a fixed hospitalization payment by the National Health Insurance to the medical institutions that are estimated based on the medical costs of previous identical or similar diseases. From January 2010 to June 2014, the first stage of this system was implemented in 164 Major Diagnostic Categories (MDCs). After the system was implemented, the average LOS decreased. There was an increase in 3-day ED visits and a 14-day readmission rate initially in 2010, which, however, declined in 2011 when the NHI administration took charge [16]. Whether the decline in ED visits and readmission rates is a result of strict control procedures described in the literature [11, 12], which may in turn increase readmission rates, or are due to delayed ED visits and readmissions to avoid indicator monitoring intentionally. Previous studies showed that possible factors for readmission and ED visits of patients include gender, age, socioeconomic status, and comorbidity [17–21]. Some DRG-related patients might be transferred to other hospitals due to cost-saving issues or other reasons. Previous studies showed that hospital transfer of critically ill patients might put patients at high risk [22], and factors that cause hospital transfers include gender, age, and comorbidity [23–25].

Many studies demonstrate that healthcare behavior is also a research emphasis in addition to healthcare quality, and they unanimously state that system implementation will cause changes in healthcare behavior [7, 26–28]. These changes may also include adverse effects. Previously, studies found that DRG-based payment systems induce morally questionable practices such as upcoding and cream-skimming, which causes an increase in the number of readmitted patients and patient selection [5, 29–33]. Concerning the effects of DRG-based payment systems on medical costs in various countries, most studies showed that total medical cost increased after system implementation [9, 34–36].

Most existing studies on DRG-based payment systems have analyzed specific diseases or their scope [9, 14]. Few studies examined the ED visits and readmission of patients in a variety of MDCs under DRG-based payment systems. Therefore, this study examines 12 MDCs in the DRG-based payment system in Taiwan from 2012 and 2013 to comprehensively analyze the distribution status and relevant factors of ED visits and readmissions of patients and the characteristics of inpatients who transferred to other hospitals and the relevant factors.

## 2. Methods

### 2.1. Data source and participants

This study is a retrospective cohort study. The data were obtained from the National Health Insurance Research Database (NHIRD) released by the National Health Research Institutes from 2012 to 2013. Inpatients in the DRG-based payment system from 1 January 2012 to 30 November 2013 were the subjects of this study, and 12 MDCs were included: MDC2, MDC3, MDC5, MDC6, MDC7, MDC8, MDC9, MDC10, MDC11, MDC12, MDC13, and MDC14. These 12 MDCs concern surgical conditions. In order to avoid subject dependence, only the first DRG hospitalization of every subject during the range of the study period was included, and data from the second hospitalization onwards were not included. It means that every sample has one set of hospitalization data within the range of the study period.

This study is mainly organized into three sections: ED visits, readmissions, and hospital transfers of inpatients under the DRG-based payment system. The first section of the analysis examined whether inpatients visited the ED within 14 days after discharge and examined the time distribution of ED visits. The second section of the analysis examined readmitted patients using 30 days after discharge as the observation period. Patients were divided into two populations depending on whether they were readmitted or not. Besides, the time distribution of readmissions of patients who were readmitted within 30 days after discharge was examined. The third section of the analysis examined hospital transfer inpatients, and the parental population was divided for comparison based on whether hospital transfers occurred.

As our study examined 30-day readmission, subjects from 1 January 2012 to 30 November 2013 were included, and subjects who were discharged after 1 December 2013 (including on that day) were excluded (*n* = 4,328). Our study also excluded patients who sought consultation at EDs in clinics (*n* = 5) and patients with missing data (*n* = 359).

### 2.2. Description of variables

In this study, the dependent variables included (1) Whether the patient visited the ED within 14 days after discharge; (2) Whether patients were readmitted within 30 days after discharge; (3) Whether hospitalized patients were transferred to other hospitals for treatment.

Independent variables included (1) patient demographic characteristics (i.e., gender, age), economic factor (i.e., monthly salary), health status (i.e., Charlson Comorbidity Index, CCI), disease diagnosis status (i.e., Major Diagnostic Categories, MDCs), emergency department triage scale, route of hospitalization (i.e., whether through the emergency department or not), and whether diagnoses were same as previous ones; (2) inpatient hospital characteristics (i.e., hospital accreditation level, hospital ownership, number of hospital beds); and (3) environmental factors (i.e., degree of urbanization of residence area, degree of urbanization of the hospital’s location).

The particular variables are described as follows: (1) The economic status of patients was measured using a monthly salary. In this study, monthly salary was classified into the ranges of ≤ 17,880 NTD (New *Taiwan Dollar*), 17,881––22,800 NTD, 22,801–28,800 NTD, 28,801–36,300 NTD, 36,301–45,800 NTD, 45,801–57,800 NTD, and ≥ 57,801 NTD. (2) For environmental factors, the level of urbanization of the place of residence was used. It was divided into Levels 1 to 7, with Level 1, the highest, and Level 7 the lowest [37]. (3) The severity of comorbidities in patients was measured using Deyo’s Charlson Comorbidity Index (CCI) modified and developed by Deyo et al. [38]. The primary diagnosis code for diseases was converted to weighted scores that were totaled to obtain the Deyo’s CCI score. In this study, the scores were divided into ranges of 0, 1, 2, and ≥ 3. (4) MDCs were the 12 categories in the DRG-based payment system from 2012 to 2013, which were: MDC2: Diseases and Disorders of the Eye, MDC3: Diseases and Disorders of the Ear, Nose, Mouth, and Throat, MDC5: Diseases and Disorders of the Circulatory System, MDC6: Diseases and Disorders of the Digestive System, MDC7: Diseases and Disorders of the Hepatobiliary System and Pancreas, MDC8: Diseases and Disorders of the Musculoskeletal System and Connective Tissue, MDC9: Diseases and Disorders of the Skin, Subcutaneous Tissue, and Breast, MDC10: Endocrine, Nutritional, and Metabolic Diseases and Disorders, MDC11: Diseases and Disorders of the Kidney and Urinary Tract, MDC12: Diseases and Disorders of the Male Reproductive System, MDC13: Diseases and Disorders of the Female Reproductive System, and MDC14: Pregnancy, Childbirth, and the Puerperium. Whether patients had the same diagnosis was determined based on the International Disease Classification Code (ICD-9-CM) diagnosis. The primary and secondary diagnoses during ED visits or readmissions were compared to see if they were identical to the primary diagnosis from the previous hospitalization stay. The emergency treatment triage scale used was the five-level Taiwan Triage and Acuity Scale released by the Ministry of Health and Welfare, with Level 1 being resuscitation, Level 2 emergency, Level 3 urgent, Level 4 less urgent, and Level 5 non-urgent [39]. (5) For inpatient hospital characteristics, hospitals were divided by accreditation level into medical centers, regional hospitals, and district hospitals. For hospital ownership, hospitals were divided into public and non-public hospitals. Hospital bed scores were divided into groups of ≤ 300 beds, 301–600 beds, 601–1000 beds, 1001–1500 beds, and ≥1501 beds.

### 2.3. Statistical analysis

Descriptive statistics were used to analyze ED visits within 14 days after discharge, the time distribution of 30-day readmissions, and whether patients were transferred to another hospital during hospitalization under the DRG-based payment system from 2012 to 2013. Independent variables were defined as described above in the description of variables.

Multilevel logistic regression was used to test the correlation between “ED visits within 14 days after discharge,” “readmission within 30 days,” and “hospital transfers” of inpatients under the DRG-based payment system with variables, including (1) individual-level such as patient characteristics, economic status, health status, disease diagnosis status, and route of hospitalization; (2) hospital-level such as hospital characteristics; (3) regional level such as environmental factors. SAS version 9.4 (SAS Institute Inc., Cary, NC, USA) statistics software was used for data processing and statistical analysis. A difference of *p* < 0.05 was considered to be statistically significant. In this study, all personal identification information was deleted, and personal privacy was protected during the use of these data. The research ethics committee approved this study at China Medical University and Hospital (IRB Number: CMU-REC-101-012).

## 3. Results

From 2012 to 2013, a total of 103,779 inpatients under the DRG-based payment system were included as study subjects (Table 1), with females accounting for most of the patients (66.72%). Most patients were from the age group of 19–44 years (49.39%) and had a CCI score of 0 (68.9%). In terms of MDCs, most patients (30.02%) had a diagnosis of Pregnancy, Childbirth, and the Puerperium (MDC14), followed by patients (25.4%) with Diseases and Disorders of the Musculoskeletal System and Connective Tissue (MDC8). Concerning the hospital accreditation level, most patients were admitted to regional hospitals (43.41%). About hospital ownership, most patients were admitted to non-public hospitals (76.76%).

**Table 1:** Baseline DRGs patient characteristics.

Variable	N	%	Variable	N	%
**Total**	**103,779**	**100.00**	MDC3	4,294	4.14
**gender**			MDC5	8,942	8.62
female	69,240	66.72	MDC6	15,285	14.73
male	34,539	33.28	MDC7	3,672	3.54
**age (year)**			MDC8	26,365	25.40
≤18	2,456	2.37	MDC9	395	0.38
19–44	51,261	49.39	MDC11	129	0.12
45–64	26,531	25.56	MDC12	1,500	1.45
≥65	23,531	22.67	MDC13	9,019	8.69
**monthly salary (NTD)**			MDC14	31,158	30.02
≤17,280	1,468	1.41	**hospital accreditation level**		
17,281–22,800	41,238	39.74	medical center	35,568	34.27
22,801–28,800	20,467	19.72	regional hospital	45,055	43.41
28,801–36,300	15,681	15.11	district hospital	23,156	22.31
36,301–45,800	11,276	10.87	**hospital ownership**		
45,801–57,800	6,078	5.86	public hospital	24,119	23.24
≥57,801	7,571	7.30	non-public hospital	79,660	76.76
**Charlson Comorbidity Index (CCI)**			**urbanization of residence area**		
0	71,506	68.90	Level 1	29,712	28.63
1	16,289	15.70	Level 2	34,272	33.02
2	8,005	7.71	Level 3	16,382	15.79
≥3	7,979	7.69	Level 4	14,008	13.50
**Major Diagnostic Category(MDC)**			Level 5	1,881	1.81
MDC10	1,878	1.81	Level 6	3,749	3.61
MDC2	1,142	1.10	Level 7	3,775	3.64

Note: The hospital is the hospital that DRGs patient discharged from.

For ED visits, we can see from Table 2 that 4,831 inpatients had ED visits within 14 days (accounting for 4.66%). Among these ED patients, 8.76% had the same primary and secondary diagnoses as on the previous hospitalization. From the bivariate analysis results in Table 2, we can see that factors, including gender, age, monthly salary, degree of urbanization of residence area, CCI, MDCs, hospital accreditation level, and hospital ownership, showed a significant correlation with ED visits within 14 days after discharge (*p* < 0.05).

**Table 2:** Bivariate and multilevel regression analysis of DRGs patients’ emergency department visits within 14 days after discharge.

Variable	No		Yes		P value	Adjusted OR	95%CI		P value
	N	%	N	%					
**Total**	98,948	95.34	4,831	4.66					
**gender**					<0.001				
female *(ref*.*)*	66,235	95.66	3,005	4.34		1.00			
male	32,713	94.71	1,826	5.29		1.00	0.93	1.08	0.952
**age (year)**					<0.001				
≤18 *(ref*.*)*	2,369	96.46	87	3.54		1.00			
19–44	49,155	95.89	2,106	4.11		1.15	0.92	1.44	0.228
45–64	25,515	96.17	1,016	3.83		0.98	0.78	1.23	0.871
≥65	21,909	93.11	1,622	6.89		1.42	1.12	1.78	0.003
**monthly salary (NTD)**					<0.001				
≤17,280*(ref*.*)*	1,342	91.42	126	8.58		1.00			
17,281–22,800	39,368	95.47	1,870	4.53		0.51	0.42	0.62	<0.001
22,801–28,800	19,446	95.01	1,021	4.99		0.59	0.48	0.71	<0.001
28,801–36,300	14,894	94.98	787	5.02		0.54	0.44	0.66	<0.001
36,301–45,800	10,836	96.10	440	3.90		0.48	0.39	0.59	<0.001
45,801–57,800	5,824	95.82	254	4.18		0.51	0.40	0.64	<0.001
≥57,801	7,238	95.60	333	4.40		0.53	0.42	0.66	<0.001
**Charlson Comorbidity Index (CCI)**					<0.001				
0 *(ref*.*)*	68,775	96.18	2,731	3.82		1.00			
1	15,469	94.97	820	5.03		1.28	1.17	1.40	<0.001
2	7,511	93.83	494	6.17		1.51	1.35	1.69	<0.001
≥3	7,193	90.15	786	9.85		2.37	2.14	2.62	<0.001
**Major Diagnostic Category(MDC)**					<0.001				
MDC10 *(ref*.*)*	1,843	98.14	35	1.86		1.00			
MDC2	1,103	96.58	39	3.42		1.23	0.77	1.97	0.389
MDC3	4,141	96.44	153	3.56		1.89	1.30	2.75	<0.001
MDC5	8,350	93.38	592	6.62		2.49	1.75	3.54	<0.001
MDC6	14,530	95.06	755	4.94		2.45	1.73	3.47	<0.001
MDC7	3,525	96.00	147	4.00		1.83	1.25	2.66	0.002
MDC8	25,063	95.06	1,302	4.94		2.11	1.50	2.98	<0.001
MDC9	387	97.97	8	2.03		0.95	0.44	2.08	0.901
MDC11	111	86.05	18	13.95		4.95	2.69	9.10	<0.001
MDC12	1,332	88.80	168	11.20		4.23	2.88	6.20	<0.001
MDC13	8,758	97.11	261	2.89		1.65	1.15	2.37	0.006
MDC14	29,805	95.66	1,353	4.34		2.66	1.88	3.77	<0.001
**hospital accreditation level**					<0.001				
medical center *(ref*.*)*	33,886	95.27	1,682	4.73		1.00			
regional hospital	42,799	94.99	2,256	5.01		1.07	0.45	2.51	0.503
district hospital	22,263	96.14	893	3.86		0.87	0.35	2.19	0.312
**hospital ownership**					<0.001				
public hospital *(ref*.*)*	22,813	94.59	1,306	5.41		1.00			
non-public hospital	76,135	95.57	3,525	4.43		0.78	0.70	0.87	<0.001
**urbanization of residence area**					<0.001				
Level 1 *(ref*.*)*	28,402	95.59	1,310	4.41		1.00			
Level 2	32,698	95.41	1,574	4.59		1.03	0.95	1.11	0.484
Level 3	15,673	95.67	709	4.33		0.98	0.89	1.08	0.711
Level 4	13,267	94.71	741	5.29		1.13	1.02	1.26	0.015
Level 5	1,797	95.53	84	4.47		0.85	0.67	1.08	0.185
Level 6	3,539	94.40	210	5.60		1.13	0.97	1.33	0.122
Level 7	3,572	94.62	203	5.38		1.11	0.95	1.31	0.189
**same diagnosis**									
No	-	-	4,408	91.24		-	-	-	-
Yes	-	-	423	8.76		-	-	-	-
**emergency triage and acuity scale**									
Level 1	-	-	139	2.88		-	-	-	-
Level 2	-	-	832	17.22		-	-	-	-
Level 3	-	-	2,722	56.34		-	-	-	-
Level 4	-	-	795	16.46		-	-	-	-
Level 5	-	-	115	2.38		-	-	-	-
unknown	-	-	228	4.72		-	-	-	-

Note: The hospital is the hospital that DRGs patient discharged from.

Multilevel logistic regression was then used to examine factors associated with ED visits within 14 days (Table 2) and identified age, monthly salary, CCI, MDC, hospital ownership, and urbanization of residence area correlated with visits (*p* < 0.05). In terms of age, patients ≤ 18 years as the reference group, the number of ED visits within 14 days was higher in patients aged ≥ 65 years old than in the reference group (OR = 1.42, 95% CI: 1.12–1.78). Patients with a monthly salary of ≤ 17,280 NTD as the reference group and the risk of ED visits within 14 days were lower in patients with higher monthly salaries than the reference group (*p* < 0.05). Patients from generally rural areas (Level 4) have a higher risk of ED visits within 14 days (OR = 1.13, 95% CI: 1.02–1.26). In terms of severity of comorbidities, the results showed that the higher the CCI score, the higher the risk of ED visits within 14 days (*p* < 0.05).

In terms of MDCs, patients with Endocrine, Nutritional, and Metabolic Diseases and Disorders (MDC10) as the reference group, patients with Diseases and Disorders of the Kidney and Urinary Tract (MDC11) had the highest risk of ED visits within 14 days (OR = 4.95, 95% CI: 2.69–9.10), followed by patients with Diseases and Disorders of the Male Reproductive System (MDC12) (OR = 4.23, 95% CI: 2.88–6.20). The risk of ED visits within 14 days was significantly lower for patients from non-public hospitals than for those from public hospitals (OR = 0.78, 95% CI: 0.70–0.87).

**Fig 1:**
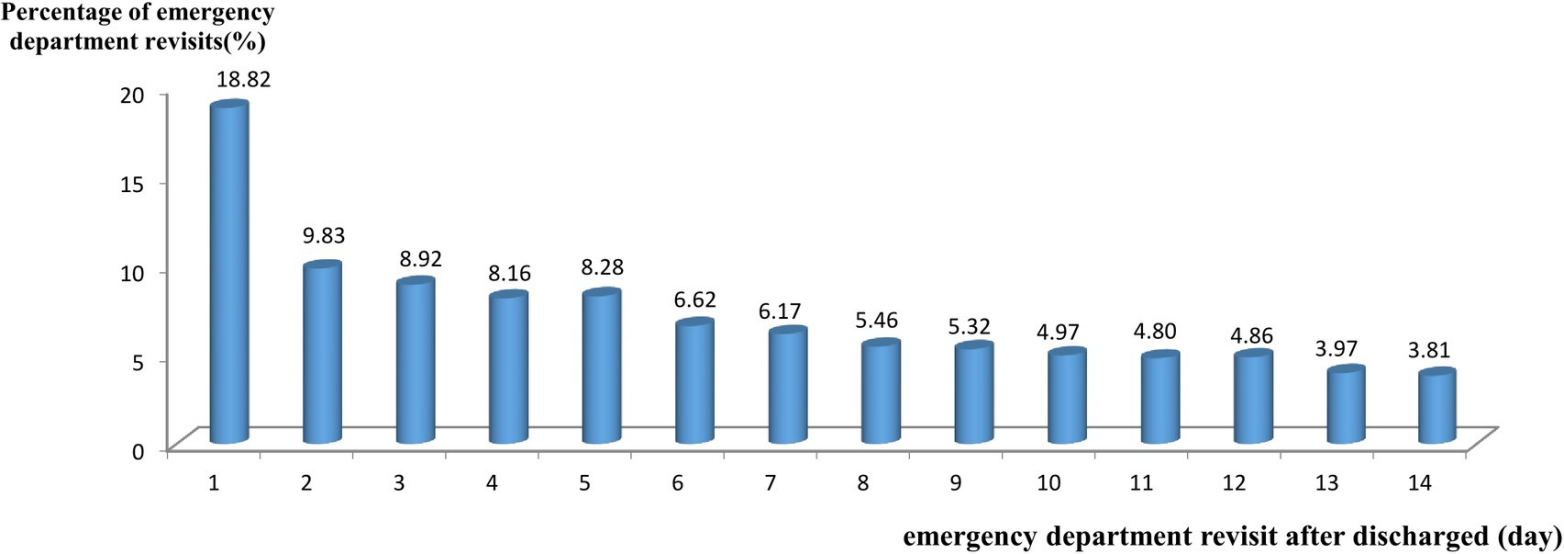
The time distribution of emergency department visits within 14 days after discharge.

We further examined the time distribution status of ED visits within 14 days after discharge and calculated the interval between discharge and ED visits by subtracting the ED visit date from the discharge date. Fig 1 shows that the proportion of patients with ED visits within one day after discharge was the highest (18.82%), which gradually decreased with the number of days. Within three days of discharge, the proportion of patients with ED visits accounted for 37.57%. Among patients visiting ED, the proportions of patients with ED visits on Day 4 (8.16%) and Day 5 (8.28%) were similar to that on Day 3 (8.92%).

Section 2 of the analysis examines whether patients were readmitted to the hospitals within 30 days of discharge. From Table 3, we can see that 7,234 patients were readmitted within 30 days (accounting for 6.97%). Among these patients, 9.28% of patients had the same primary and secondary diagnoses as the previous hospitalization. From the bivariate analysis results in Table 3, we can see that gender, age, monthly salary, CCI, MDCs, hospital accreditation level, and hospital ownership show a significant correlation with readmission within 30 days of discharge (*p* < 0.05).

**Table 3:** Bivariate and multilevel regression analysis of DRGs patients’ readmissions within 30 days after discharge.

Variable	Total		No		Yes		P value	Adjusted OR	95%CI		P value
	N	%	N	%	N	%					
**Total**	103,779	100.00	96,545	93.03	7,234	6.97					
**gender**							<0.001				
female *(ref*.*)*	69,240	66.72	63,832	92.19	5,408	7.81		1.00			
male	34,539	33.28	32,713	94.71	1,826	5.29		1.28	1.18	1.39	<0.001
**age (year)**							<0.001				
≤18 *(ref*.*)*	2,456	2.37	2,397	97.60	59	2.40		1.00			
19–44	51,261	49.39	46,888	91.47	4,373	8.53		1.35	1.02	1.77	0.033
45–64	26,531	25.56	25,512	96.16	1,019	3.84		1.69	1.29	2.23	<0.001
≥65	23,531	22.67	21,748	92.42	1,783	7.58		2.54	1.93	3.35	<0.001
**monthly salary (NTD)**							<0.001				
≤17,280 *(ref*.*)*	1,468	1.41	1,301	88.62	167	11.38		1.00			
17,281–22,800	41,238	39.74	38,425	93.18	2,813	6.82		0.48	0.40	0.57	<0.001
22,801–28,800	20,467	19.72	19,109	93.36	1,358	6.64		0.48	0.40	0.58	<0.001
28,801–36,300	15,681	15.11	14,523	92.62	1,158	7.38		0.50	0.42	0.60	<0.001
36,301–45,800	11,276	10.87	10,508	93.19	768	6.81		0.48	0.40	0.58	<0.001
45,801–57,800	6,078	5.86	5,605	92.22	473	7.78		0.45	0.37	0.54	<0.001
≥57,801	7,571	7.30	7,074	93.44	497	6.56		0.48	0.39	0.58	<0.001
**Charlson Comorbidity Index (CCI)**							<0.001				
0 *(ref*.*)*	71,506	68.90	66,726	93.32	4,780	6.68		1.00			
1	16,289	15.70	15,342	94.19	947	5.81		1.19	1.10	1.29	<0.001
2	8,005	7.71	7,441	92.95	564	7.05		1.58	1.42	1.75	<0.001
≥3	7,979	7.69	7,036	88.18	943	11.82		2.62	2.38	2.89	<0.001
**Major Diagnostic Category (MDC)**							<0.001				
MDC10 *(ref*.*)*	1,878	1.81	1,855	98.78	23	1.22		1.00			
MDC2	1,142	1.10	1,055	92.38	87	7.62		3.33	2.06	5.37	<0.001
MDC3	4,294	4.14	4,231	98.53	63	1.47		1.13	0.69	1.84	0.628
MDC5	8,942	8.62	8,093	90.51	849	9.49		4.45	2.91	6.82	<0.001
MDC6	15,285	14.73	14,839	97.08	446	2.92		1.87	1.22	2.87	0.004
MDC7	3,672	3.54	3,555	96.81	117	3.19		1.98	1.26	3.13	0.003
MDC8	26,365	25.40	24,984	94.76	1,381	5.24		3.05	2.00	4.66	<0.001
MDC9	395	0.38	351	88.86	44	11.14		9.53	5.62	16.13	<0.001
MDC11	129	0.12	116	89.92	13	10.08		3.98	1.93	8.20	<0.001
MDC12	1,500	1.45	1,397	93.13	103	6.87		2.52	1.58	4.04	<0.001
MDC13	9,019	8.69	8,875	98.40	144	1.60		1.64	1.04	2.57	0.032
MDC14	31,158	30.02	27,194	87.28	3,964	12.72		20.43	13.32	31.34	<0.001
**hospital accreditation level**							<0.001				
medical center *(ref*.*)*	35,568	34.27	33,425	93.97	2,143	6.03		1.00			
regional hospital	45,055	43.41	41,379	91.84	3,676	8.16		1.25	0.23	6.69	0.339
district hospital	23,156	22.31	21,741	93.89	1,415	6.11		0.87	0.17	4.59	0.485
**hospital ownership**							0.036				
public hospital *(ref*.*)*	24,119	23.24	22,511	93.33	1,608	6.67		1.00			
non-public hospital	79,660	76.76	74,034	92.94	5,626	7.06		0.80	0.67	0.96	0.019
**urbanization of residence area**							0.147				
Level 1 *(ref*.*)*	29,712	28.63	27,666	93.11	2,046	6.89		1.00			
Level 2	34,272	33.02	31,887	93.04	2,385	6.96		0.99	0.93	1.06	0.855
Level 3	16,382	15.79	15,292	93.35	1,090	6.65		1.02	0.94	1.11	0.683
Level 4	14,008	13.50	13,009	92.87	999	7.13		1.01	0.92	1.11	0.828
Level 5	1,881	1.81	1,745	92.77	136	7.23		1.15	0.94	1.39	0.168
Level 6	3,749	3.61	3,460	92.29	289	7.71		1.14	0.99	1.32	0.073
Level 7	3,775	3.64	3,486	92.34	289	7.66		1.11	0.96	1.28	0.172
**readmission with same diagnosis**											
No					6,563	90.72		-	-	-	-
Yes					671	9.28		-	-	-	-

Note: The hospital was the hospital that DRGs patients discharged from.

We employed multilevel logistic regression to examine factors associated with readmission within 30 days (Table 3). The results show that gender, age, monthly salary, CCI, MDCs, and hospital ownership showed significant correlations with readmission within 30 days of discharge (*p* < 0.05). In terms of gender, the risk of male patients being readmitted within 30 days of discharge was significantly higher than in female patients (OR = 1.28, 95% CI: 1.18–1.39). Patients ≤ 18 years used as the reference group. The patients who were older than the reference group had a significantly higher risk of readmission within 30 days (*p* < 0.05), and the risk of readmission within 30 days increased with age. In terms of monthly salary, patients with a monthly salary of ≤ 17,280 NTD taken as the reference group, the risk of readmission within 30 days is significantly lower in patients with higher monthly salaries than the reference group (*p* < 0.05). In terms of severity of comorbidities, results showed that the higher the CCI score, the higher the risk of readmission within 30 days (*p* < 0.05). In terms of MDCs, patients with Endocrine, Nutritional, and Metabolic Diseases and Disorders (MDC10) used as the reference group, as they had the lowest proportion of readmission within 30 days, results showed that patients with Pregnancy, Childbirth, and the Puerperium (MDC14) had the highest risk of readmission within 30 days (OR = 20.43, 95%CI: 13.32–31.34), followed by patients with Diseases and Disorders of the Skin, Subcutaneous Tissue, and Breast (MDC9) (OR = 9.53, 95% CI: 5.62–16.13). Public hospitals taken as the reference group, patients from non-public hospitals had a significantly lower risk of readmission within 30 days (OR = 0.80, 95%CI:0.67–0.96).

**Fig 2:**
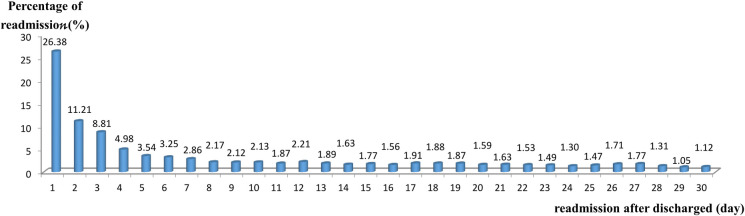
The time distribution of readmission within 30 days after discharge.

We further examined the time distribution of readmission within 30 days of discharge and calculated the interval between discharge and readmission by subtracting the readmission date from the discharge date. From Fig 2, we can see that the proportion of patients with readmission within one day after discharge was the highest (26.38%), which gradually decreased with the number of days. Within 14 days of discharge, the proportion of patients with readmission accounted for 75.05%.

Section 3 of the analysis examined patients who underwent hospital transfers. From Table 4, we can see that out of 103,779 inpatients, only 169 inpatients transferred hospitals (accounting for 0.16%). From the bivariate analysis in Table 4, we can see that gender, age, urbanization of residence area, urbanization of the hospital’s location, CCI, MDCs, hospital accreditation level, number of hospital beds, and route of hospitalization were significantly correlated with whether patients underwent hospital transfers (*p* < 0.05).

**Table 4:** Bivariate and multilevel regression analysis of DRGs patients’ hospital transfer.

Variable	Total		No		Yes		P value	Adjusted OR	95%CI		P value
	N	%	N	%	N	%					
**Total**	103,779	100.00	103,610	99.84	169	0.16					
**gender**							0.030				
female *(ref*.*)*	69,240	66.72	69,141	99.86	99	0.14		1.00			
male	34,539	33.28	34,469	99.80	70	0.20		1.74	1.13	2.70	0.013
**age (year)**							0.011				
≤18 *(ref*.*)*	2,456	2.37	2,455	99.96	1	0.04		1.00			
19–44	51,261	49.39	51,184	99.85	77	0.15		2.37	0.31	17.91	0.402
45–64	26,531	25.56	26,495	99.86	36	0.14		2.23	0.30	16.81	0.435
≥65	23,531	22.67	23,476	99.77	55	0.23		2.55	0.34	19.24	0.365
**monthly salary (NTD)**							0.818				
≤17,280 *(ref*.*)*	1,468	1.41	1,466	99.86	2	0.14		1.00			
17,281–22,800	41,238	39.74	41,173	99.84	65	0.16		1.20	0.29	4.98	0.801
22,801–28,800	20,467	19.72	20,429	99.81	38	0.19		1.67	0.40	7.04	0.482
28,801–36,300	15,681	15.11	15,653	99.82	28	0.18		1.44	0.34	6.14	0.626
36,301–45,800	11,276	10.87	11,256	99.82	20	0.18		1.84	0.42	8.03	0.416
45,801–57,800	6,078	5.86	6,071	99.88	7	0.12		1.17	0.24	5.75	0.848
≥57,801	7,571	7.30	7,562	99.88	9	0.12		1.41	0.30	6.66	0.664
**Charlson Comorbidity Index (CCI)**							<0.001				
0 *(ref*.*)*	71,506	68.90	71,420	99.88	86	0.12		1.00			
1	16,289	15.70	16,261	99.83	28	0.17		1.55	0.95	2.51	0.077
2	8,005	7.71	7,979	99.68	26	0.32		2.88	1.68	4.96	<0.001
≥3	7,979	7.69	7,950	99.64	29	0.36		2.45	1.40	4.30	0.002
**Major Diagnostic Category (MDC)**							<0.001				
MDC13 *(ref*.*)*	9,019	8.69	9,017	99.98	2	0.02		1.00			
MDC2	1,142	1.10	1,141	99.91	1	0.09		-	-	-	-
MDC3	4,294	4.14	4,294	100.00	0	0.00		-	-	-	-
MDC5	8,942	8.62	8,893	99.45	49	0.55		9.51	2.09	43.19	0.004
MDC6	15,285	14.73	15,279	99.96	6	0.04		0.60	0.11	3.13	0.540
MDC7	3,672	3.54	3,669	99.92	3	0.08		1.48	0.24	9.26	0.676
MDC8	26,365	25.40	26,321	99.83	44	0.17		1.95	0.44	8.66	0.377
MDC9	395	0.38	395	100.00	0	0.00		-	-	-	-
MDC10	1,878	1.81	1,878	100.00	0	0.00		-	-	-	-
MDC11	129	0.12	129	100.00	0	0.00		-	-	-	-
MDC12	1,500	1.45	1,499	99.93	1	0.07		-	-	-	-
MDC14	31,158	30.02	31,095	99.80	63	0.20		5.61	1.32	23.83	0.020
**whether through the ED or not**							<0.001				
No *(ref*.*)*	77,690	74.86	77,594	99.88	96	0.12		1.00			
Yes	26,089	25.14	26,016	99.72	73	0.28		2.42	1.73	3.39	<0.001
**hospital accreditation level**							<0.001				
medical center *(ref*.*)*	35,496	34.20	35,478	99.95	18	0.05		1.00			
regional hospital	45,089	43.45	44,993	99.79	96	0.21		1.94	0.01	543.05	0.375
district hospital	23,194	22.35	23,139	99.76	55	0.24		1.13	0.00	3342.59	0.874
**hospital ownership**							0.892				
public hospital *(ref*.*)*	24,099	23.22	24,061	99.84	38	0.16		1.00			
non-public hospital	79,680	76.78	79,549	99.84	131	0.16		0.86	0.52	1.42	0.547
**number of hospital beds**							<0.001				
≤300 beds *(ref*.*)*	18,692	18.01	18,645	99.75	47	0.25		1.00			
301–600 beds	17,171	16.55	17,118	99.69	53	0.31		0.51	0.17	1.47	0.174
601–1,000 beds	23,081	22.24	23,040	99.82	41	0.18		0.25	0.07	0.88	0.035
1,001–1,500 beds	23,553	22.70	23,533	99.92	20	0.08		0.15	0.04	0.60	0.014
≥1,501 beds	21,282	20.51	21,274	99.96	8	0.04		0.09	0.01	0.58	0.018
**urbanization of residence area**							<0.001				
Level 1 *(ref*.*)*	29,712	28.63	29,683	99.90	29	0.10		1.00			
Level 2	34,272	33.02	34,204	99.80	68	0.20		1.44	0.90	2.30	0.123
Level 3	16,382	15.79	16,365	99.90	17	0.10		0.83	0.44	1.55	0.556
Level 4	14,008	13.50	13,972	99.74	36	0.26		1.64	0.94	2.85	0.080
Level 5	1,881	1.81	1,876	99.73	5	0.27		1.94	0.70	5.33	0.200
Level 6	3,749	3.61	3,743	99.84	6	0.16		1.15	0.45	2.93	0.771
Level 7	3,775	3.64	3,767	99.79	8	0.21		1.30	0.56	3.03	0.544
**urbanization of hospital’s location**							<0.001				
Level 1 *(ref*.*)*	33,833	32.60	33,809	99.93	24	0.07		1.00			
Level 2	49,153	47.36	49,054	99.80	99	0.20		1.77	0.98	3.20	0.059
Level 3	8,154	7.86	8,141	99.84	13	0.16		1.48	0.63	3.46	0.363
Level 4	11,474	11.06	11,442	99.72	32	0.28		1.71	0.83	3.54	0.146
Level 5–7	1,165	1.12	1,164	99.91	1	0.09		0.50	0.06	4.22	0.519

Note: The hospital was the hospital that DRGs patient discharged from. Logistic regression model did not include MDC2, MDC3, MDC9, MDC10, MDC11, and MDC12 patients.

We employed multilevel logistic regression to examine factors associated with hospital transfers (Table 4). Results showed that gender, CCI, MDCs, number of hospital beds, and hospitalization route showed significant correlations with hospital transfers (*p* < 0.05). Regarding gender, the risk of hospital transfer in male patients was significantly higher than in female patients (OR = 1.74, 95%CI: 1.13–2.70). In terms of severity of comorbidities, patients with a CCI score of 2 points (OR = 2.88, 95%CI: 1.68–4.96) or ≥ 3 points (OR = 2.45, 95%CI: 1.40–4.30) had the highest risk of hospital transfers. Concerning MDCs, disease categories without hospital transfers (MDC 2, 3, 9, 10, 11, and 12) were excluded. Patients with Diseases and Disorders of the Circulatory System (MDC5) had the highest risk of hospital transfer (OR = 9.51, 95% CI: 2.09–43.19), followed by patients with Pregnancy, Childbirth, and the Puerperium (MDC14) (OR = 5.61, 95%CI: 1.32–23.83). In terms of the number of hospital beds, patients from hospitals with ≥ 601 beds had a significantly lower risk of hospital transfer (*p* < 0.05), and overall patients from hospitals with ≥ 1501 beds had the lowest risk of hospital transfer (OR = 0.09, 95%CI: 0.01–0.58). In terms of route of hospitalization, patients who were admitted through the ED had a significantly higher risk of hospital transfer (OR = 2.36, 95%CI: 1.71–3.27).

## 4. Discussion

This study examined the patient characteristics of ED visits within 14 days and the relevant factors for such patients under the DRG-based payment system. Concerning patient characteristics, the results in Table 2 show that patients ≥ 65 years old had the highest risk of ED visits within 14 days. It may be because elderly patients are prone to careless falls [40], poor health status, or chronic diseases, which increase the rate of ED visits. Our study showed that patients with a higher monthly salary had a lower risk of ED visits within 14 days. A previous US study examined patients who underwent septorhinoplasty [20]. The results showed that patients with lower socioeconomic status had a higher risk of ED visits within 30 days [20], which is similar to the results of this study. Turning to the severity of comorbidities, the results of this study showed that the risk of ED visits within 14 days increased as the CC1 score of patients increased, of whom patients with CCI ≥ 3 points had the highest risk of ED visits within 14 days. It is consistent with the results of a previous US study that examined the risk of ED visits within three days, seven days, 30 days, and one year in patients aged ≥ 65. The results showed that the higher the CCI score of the patient, the higher the risk of ED visits [40].

About disease diagnosis categories, the results of this study showed that patients with Diseases and Disorders of the Kidney and Urinary Tract (MDC11) had the highest risk of ED visits within 14 days, followed by patients with Diseases and Disorders of the Male Reproductive System (MDC12). A previous study in Taiwan examined patients who had ED visits within three days and showed that patients with diseases of the digestive system accounted for most patients with ED visits [41], which is not consistent with the results of this study. This discrepancy in reporting could be because Diseases and Disorders of the Digestive System (MDC6) in our study included only surgical patients. In contrast, the study subjects in that paper included internal medicine and surgical patients, resulting in differences in the proportion of patients in disease categories. The results of this study show that among patients who had ED visits within 14 days, most patients had a triage grade of 3 (accounting for 56.34%), while the proportion of patients with triage grades 4 and 5 (18.84%) was similar to that of patients with grades 1 and 2 (20.1%). This result is similar to that of a Canadian study that found that patients with ED visits are mostly less urgent patients and not urgent ones [42].

With regard to the risk of readmission within 30 days of discharge, the results of this study show that male patients had a significantly higher risk than female patients. It is similar to the findings of the previous study in the US of patients with inflammatory bowel disease that examined the characteristics of patients who were readmitted within 30 days [43]. It showed that the risk of readmission within 30 days was higher in males than in female patients [43]. With regard to age, the results of this study showed that patients aged ≥ 65 years old had the highest risk of readmission within 30 days. A previous US study examined the revisit rate (including readmission rate for outpatient procedures, readmission rate, or ED revisit) in patients who underwent septorhinoplasty [20]. Results showed that patients aged ≥ 65 years old had a higher risk of hospital revisit rate [20], which is similar to the results of this study. Concerning monthly salaries, the results of this study showed that patients with higher monthly salaries had a lower risk of readmission within 30 days. A study of patients with inflammatory bowel disease examined the characteristics of patients who were readmitted within 30 days [43] and found that patients with a higher mean income had a lower risk of readmission than patients with a lower mean income is similar to the results of this study.

Concerning the severity of comorbidities, the results of this study showed that patients with CCI ≥ 3 points had the highest risk of readmission within 30 days. A previous US study of patients who underwent colectomy showed that patients with CCI ≥ 2 points had a significantly higher risk of readmission within 30 days [17], which is similar to the results of this study. About disease diagnosis categories, our study found that patients with Pregnancy, Childbirth, and the Puerperium (MDC14) had the highest risk of readmission within 30 days. It may be because pregnancy, childbirth, and puerperium are unstable periods, and patients may be hospitalized for short periods for delivery. Therefore, MDC14 patients have a higher risk of readmission than do patients in other disease diagnosis categories. However, previous studies found that under the DRG-based payment system, the risk of readmission within 30 days in patients who underwent specific obstetric and gynecological surgeries (cesarean section, hysterectomy, etc.) showed a decreasing trend [44]. A previous study in France examined the risk of readmission within 30 days and the trends in patients from different MDCs before and after implementing a DRG-based payment system [45]. They found that as time progressed, ophthalmology gradually became the disease category with the highest risk of readmission within 30 days, while obstetric and gynecological diseases also had a higher risk of readmission within 30 days than other disease categories [45]. Our study also found that patients with Diseases and Disorders of the Skin, Subcutaneous Tissue, and Breast (MDC9) had the second-highest risk of readmission within 30 days. We conducted further analyses and found that a primary reason was that the unilateral total or subtotal mastectomy for carcinoma in situ of the breast without complication and comorbidity had a higher readmission rate. A previous study showed a high rate of incomplete resection in extensive ductal carcinoma in situ (DCIS) of breast cancer, which led to readmission for surgery [46].

In Taiwan, in order to prevent doctors’ dividing up DRG-based hospital stays for seeking health insurance reimbursement, the NHI monitored readmissions and would not reimburse for the second admission within 14 days after discharge. From Fig 2, which shows the time distribution of readmission within 30 days of discharge, we can see that patients admitted within 14 days after discharge accounted for 75.03% of all readmissions. A small increase in the number of patients readmitted on day 15 (1.77%) was not significantly different from those who had a readmission on day 14 (1.63%). Thus, from this study, it was not apparent that healthcare providers displayed rule avoidance and advantage-taking behaviors as has been mentioned in previous literature as occurring when DRG-based payment systems were implemented in some countries [29–31].

There has been no study examining the relevant factors for hospital transfer in patients under the DRG-based payment system, and our study can be considered the first paper to do so. The results of this study's analysis show that the risk of hospital transfer in male patients is significantly higher than that of female patients. A previous US study of the relevant factors for hospital transfer in hospitalized patients across the US showed that male patients have a higher risk of hospital transfer than female patients [25], which is similar to the results of this study. It may be because of innate differences in the risk of developing diseases between males and females [47]. We further conducted stratification analyses according to gender among patients who were transferred to other hospitals and found that among this group, male patients were more likely to be older than 45 years (68.14% vs 39.62%) with a higher Charlson comorbidity index as compared to their female counterparts. Besides, 32.68% of male patients were diagnosed with MDC8, which had a higher hospital transfer rate, whereas 21.77% of female patients diagnosed with this category of disease. It is why male patients had a higher risk of hospital transfer than in female patients.

With regard to the severity of comorbidities, the results of this study showed that patients with CCI ≥ 3 points had a higher risk of hospital transfer. A study of patients ≥ 65 years old examined their risk of hospital transfer and showed that patients with higher CCI had a higher risk of hospital transfer [48]. Concerning MDCs, no previous study has compared the correlation between MDCs and the risk of hospital transfer. This study shows that patients with Diseases and Disorders of the Circulatory System (MDC5) had the highest risk of a hospital transfer, followed by patients with Pregnancy, Childbirth, and the Puerperium (MDC14). It is because patients with MDC5 may be emergency patients, and immediate hospital transfer for treatment is required if the hospital is unable to treat the patient immediately, resulting in these patients having a higher risk of hospital transfer than those in other categories. On the other hand, due to emergency, MDC14 patients may be admitted for surgeries to neighboring hospitals that are not those they usually visit for a consultation. Therefore, this may cause patients to transfer to other hospitals after delivery, resulting in a higher risk of hospital transfer.

Moreover, there was no study in the past, examining the correlation between hospital beds and the risk of hospital transfer. Our analysis results showed that patients from hospitals with ≥ 601 beds had a significantly lower risk of a hospital transfer. Patients from hospitals with ≥ 1501 beds had the lowest risk of hospital transfer. This phenomenon is consistent with general reasoning: As the number of beds increases, the hospital has a higher capacity to treat patients. The rate of hospital transfer is relatively lower. Therefore, it can be observed that the risk of hospital transfer decreases as the number of beds increases. About the route of hospitalization, few studies have examined whether the risk of hospital transfer is related to admission through the ED. The results of this study show that patients who were admitted through the ED had a higher risk of hospital transfer. It may be because the hospital where a patient was admitted through the ED for hospitalization and treatment was not the hospital where the patient usually seeks consultation. It results in a higher risk to the patient of hospital transfer during hospitalization.

## 5. Limitations

Our study was unable to accurately compare the case payment classification method that was implemented before the DRG-based payment system and the classification methods in the DRG-based payment system. Therefore, we were unable to examine the differences before and after implementing the DRG-based payment system. Besides, we found from our literature review that many factors are associated with readmission or ED visits. However, some data can only be obtained from questionnaire surveys. As our study is an analysis of the NHIRD, we were unable to include all possible factors for a complete examination. Concerning the readmission of study subjects, we were unable to distinguish between planned and unplanned readmissions. Therefore, we were unable to examine patients with unplanned readmissions independently. During the literature review, we found that physician availability is one of the critical factors for ED visits or readmissions by patients. However, as the limited physician data in the NHIRD, we, therefore, did not include physician factors in this study. Finally, since our patients were mainly surgical patients, the study findings cannot be generalized to medical admissions.

## 6. Conclusions

Our study found that under the DRG-based payment system, MDCs are important correlation factors for ED visits within 14 days, readmission within 30 days, or hospital transfers. This study showed that patients with Diseases and Disorders of the Kidney and Urinary Tract (MDC11) had the highest risk of ED visits within 14 days, followed by patients with Diseases and Disorders of the Male Reproductive System (MDC12). About the risk of readmission within 30 days, patients with Pregnancy, Childbirth, and the Puerperium (MDC14) had the highest risk, followed by patients with Diseases and Disorders of the Skin, Subcutaneous Tissue, and Breast (MDC9). Concerning hospital transfer, patients with Diseases and Disorders of the Circulatory System (MDC5) had the highest risk of hospital transfers, followed by patients with Pregnancy, Childbirth, and the Puerperium (MDC14). We recommend further investigation into the possible reasons for the higher risk of ED visits, readmission, and hospital transfers for patients in the MDCs that have a higher risk of such actions. Finally, we did not find healthcare providers had significant rule avoidance and advantage-taking behaviors, in which hospitals may delay readmission by 1 to 3 days to avoid the 14-day readmission indicator.

## List of abbreviations

DRGs: Diagnosis Related Groups
MDC: Major Diagnostic Category
NHI: National Health Insurance
NHIRD: National Health Insurance Research Database
ICD-9-CM: International Classification of Diseases, Ninth Revision, Clinical Modification
CCI: Charlson Comorbidity Index
ED: Emergency department
OR: odds ratio
CI: confidence interval
PPS: Prospective payment system
NTD: New Taiwan dollar
IRB: institution review board
SSTI: skin and soft tissue infection

## References

[1] Stern RS, Epstein AM. Institutional responses to prospective payment based on diagnosis-related groups. Implications for cost, quality, and access. The New England journal of medicine. 1985;312(10):621–7. Epub 1985/03/07. 10.1056/NEJM198503073121005 . 3919294

[2] Moreno-Serra R, Wagstaff A. System-wide impacts of hospital payment reforms: evidence from Central and Eastern Europe and Central Asia. Journal of health economics. 2010;29(4):585–602. Epub 2010/06/23. 10.1016/j.jhealeco.2010.05.007 . 20566226

[3] Schargus M, Gass P, Neubauer A, Kotas M. Development of the German diagnosis-related groups (G-DRG) for ophthalmology from 2004 to 2012. Der Ophthalmologe: Zeitschrift der Deutschen Ophthalmologischen Gesellschaft. 2014;111(4):354–64. Epub 2013/06/20. 10.1007/s00347-013-2886-y . 23779251

[4] Jang SI, Nam CM, Lee SG, Kim TH, Park S, Park EC. Impact of payment system change from per-case to per-diem on high severity patient's length of stay. Medicine. 2016;95(37). 10.1097/MD.0000000000004839 27631239 PMC5402582

[5] Wang K, Li P, Chen L, Kato K, Kobayashi M, Yamauchi K. Impact of the Japanese diagnosis procedure combination-based payment system in Japan. Journal of medical systems. 2010;34(1):95–100. Epub 2010/03/03. 10.1007/s10916-008-9220-2 . 20192060

[6] Latta VB, Helbing C. Medicare: short-stay hospital services, by leading diagnosis-related groups, 1983 and 1985. Health care financing review. 1988;10(2):79–107. Epub 1988/12/05. 10313089 PMC4192922

[7] Cheng SH, Chen CC, Tsai SL. The impacts of DRG-based payments on health care provider behaviors under a universal coverage system: a population-based study. Health policy (Amsterdam, Netherlands). 2012;107(2–3):202–8. Epub 2012/04/27. 10.1016/j.healthpol.2012.03.021 . 22534586

[8] Kim H, Jung IM, Yun KW, Heo SC, Ahn YJ, Hwang K-T, et al Early outcome of the Korean Diagnosis-Related Groups payment system for appendectomy. Ann Surg Treat Res. 2015;88(3):126–32. 10.4174/astr.2015.88.3.126 25741491 PMC4347045

[9] Moon SB. Early results of pediatric appendicitis after adoption of diagnosis-related group-based payment system in South Korea. Journal of Multidisciplinary Healthcare. 2015;8:503–9. 10.2147/JMDH.S95937 26648734 PMC4664545

[10] Mihailovic N, Kocic S, Jakovljevic M. Review of Diagnosis-Related Group-Based Financing of Hospital Care. Health services research and managerial epidemiology. 2016;3:2333392816647892 Epub 2017/05/04. 10.1177/2333392816647892 28462278 PMC5266471

[11] Ishii M. DRG/PPS and DPC/PDPS as Prospective Payment Systems. Japan Medical Association journal: JMAJ. 2012;55(4):279–91. Epub 2012/07/01. . 25237234

[12] Hamada H, Sekimoto M, Imanaka Y. Effects of the per diem prospective payment system with DRG-like grouping system (DPC/PDPS) on resource usage and healthcare quality in Japan. Health policy (Amsterdam, Netherlands). 2012;107(2–3):194–201. Epub 2012/01/27. 10.1016/j.healthpol.2012.01.002 . 22277879

[13] Forgione DA, Vermeer TE, Surysekar K, Wrieden JA, Plante CA. The impact of DRG-based payment systems on quality of health care in OECD countries. Journal of health care finance. 2004;31(1):41–54. Epub 2005/04/09. . 15816228

[14] Ljunggren B, Sjödén PO. Patient reported quality of care before vs. after the implementation of a diagnosis related groups (DRG) classification and payment system in one Swedish county. Scandinavian Journal of Caring Sciences. 2001;15(4):283–94. 10.1046/j.1471-6712.2001.00046.x 12453169

[15] Busato A, von Below G. The implementation of DRG-based hospital reimbursement in Switzerland: A population-based perspective. Health research policy and systems. 2010;8:31 Epub 2010/10/19. 10.1186/1478-4505-8-31 20950481 PMC2973930

[16] National Health Insurance Administration, Ministry of Health and Welfare. National Health Insurance Annual Report (2016–2017) Accessed June 14, 2017. Available from: https://www.nhi.gov.tw/Content_List.aspx?n=F02F2F627FE4BEC4&topn=CDA985A80C0DE710.

[17] Kulaylat AN, Dillon PW, Hollenbeak CS, Stewart DB. Determinants of 30-d readmission after colectomy. The Journal of surgical research. 2015;193(2):528–35. Epub 2014/12/03. 10.1016/j.jss.2014.09.029 . 25438957

[18] Rubin DJ. Hospital readmission of patients with diabetes. Current diabetes reports. 2015;15(4):17 Epub 2015/02/26. 10.1007/s11892-015-0584-7 . 25712258

[19] Ansari SF, Yan H, Zou J, Worth RM, Barbaro NM. Hospital Length of Stay and Readmission Rate for Neurosurgical Patients. Neurosurgery. 2017 Epub 2017/04/13. 10.1093/neuros/nyx160 . 28402465

[20] Spataro E, Branham GH, Kallogjeri D, Piccirillo JF, Desai SC. Thirty-Day Hospital Revisit Rates and Factors Associated With Revisits in Patients Undergoing Septorhinoplasty. JAMA facial plastic surgery. 2016;18(6):420–8. 10.1001/jamafacial.2016.0539 27311117 PMC5600887

[21] Wang KC, Chaou CH, Liu PH, Chien CY, Lee CH. Factors Affecting Unscheduled Return Visits to the Emergency Department among Minor Head Injury Patients. BioMed Research International. 2017;2017:8963102 Epub 2017/10/12. 10.1155/2017/8963102 29018821 PMC5605872

[22] Doherty P, Digby B. Analysis of critical incidents during the interhospital transport of critically ill patients: Crit Care. 2007;11(Suppl 2):P502 Epub 2007 Mar 22 10.1186/cc5662; 2007.

[23] Odetola FO, Clark SJ, Gurney JG, Donohue JE, Gebremariam A, Allerton L, et al Factors Associated with Inter-hospital Transfer of Children in Respiratory Failure from Level II to Level I Pediatric Intensive Care Units. Journal of critical care. 2015;30(5):1080–4. 10.1016/j.jcrc.2015.06.008 PMC4681687. 26117217 PMC4681687

[24] Westfall JM, Kiefe CI, Weissman NW, Goudie A, Centor RM, Williams OD, et al Does interhospital transfer improve outcome of acute myocardial infarction? A propensity score analysis from the Cardiovascular Cooperative Project. BMC Cardiovascular Disorders. 2008;8:22 10.1186/1471-2261-8-22 18782452 PMC2551582

[25] Hernandez-Boussard T, Davies S, McDonald K, Wang NE. Interhospital Facility Transfers in the United States: A Nationwide Outcomes Study. Journal of patient safety. 2017;13(4):187–91. 10.1097/PTS.0000000000000148 25397857 PMC4956577

[26] Hensen P, Beissert S, Bruckner-Tuderman L, Luger TA, Roeder N, Müller ML. Introduction of diagnosis-related groups in Germany: evaluation of impact on in-patient care in a dermatological setting. European Journal of Public Health. 2008;18(1):85–91. 10.1093/eurpub/ckm059 17569699

[27] Kim SJ, Han KT, Kim SJ, Park EC, Park HK. Impact of a diagnosis-related group payment system on cesarean section in Korea. Health policy (Amsterdam, Netherlands). 2016;120(6):596–603. Epub 2016/05/14. 10.1016/j.healthpol.2016.04.018 . 27173768

[28] Hu WY, Yeh CF, Shiao AS, Tu TY. Effects of diagnosis-related group payment on health-care provider behaviors: A consecutive three-period study. Journal of the Chinese Medical Association: JCMA. 2015;78(11):678–85. Epub 2015/09/06. 10.1016/j.jcma.2015.06.012 . 26341451

[29] Berta P, Callea G, Martini G, Vittadini G. The effects of upcoding, cream skimming and readmissions on the Italian hospitals efficiency: a population-based investigation. Economic Modelling. 2010;27(4):812–21.

[30] Jurges H, Koberlein J. What explains DRG upcoding in neonatology? The roles of financial incentives and infant health. Journal of health economics. 2015;43:13–26. Epub 2015/06/27. 10.1016/j.jhealeco.2015.06.001 . 26114589

[31] Radu CP, Chiriac DN, Vladescu C. Changing Patient Classification System for Hospital Reimbursement in Romania. Croatian Medical Journal. 2010;51(3):250–8. 10.3325/cmj.2010.51.250 20564769 PMC2897082

[32] Nakamura K. Development of the reimbursement system based on DPC. Rinsho byori The Japanese journal of clinical pathology. 2004;52(11):906–14. Epub 2005/01/22. . 15658470

[33] Gaughan J, Kobel C. Coronary artery bypass grafts and diagnosis related groups: patient classification and hospital reimbursement in 10 European countries. Health Economics Review. 2014;4:4 10.1186/s13561-014-0004-8 24949279 PMC4052634

[34] Jacobs VR, Rasche L, Harbeck N, Warm M, Mallmann P. Underfinancing of 90.3% for implant costs of prostheses and expanders in DRG revenues for uni- and bilateral mastectomy with immediate breast reconstruction. Onkologie. 2010;33(11):584–8. Epub 2010/10/27. 10.1159/000321144 . 20975304

[35] Lotter O, Stahl S, Beck M, Loewe W, Schaller HE. Development of diagnosis-related groups in different surgical disciplines. Zentralblatt fur Chirurgie. 2014;139 Suppl 2:e109–15. Epub 2011/06/21. 10.1055/s-0031-1271532 . 21688237

[36] Kim SJ, Han KT, Kim W, Kim SJ, Park EC. Early Impact on Outpatients of Mandatory Adoption of the Diagnosis-Related Group-Based Reimbursement System in Korea on Use of Outpatient Care: Differences in Medical Utilization and Presurgery Examination. Health services research. 2017 Epub 2017/08/15. 10.1111/1475-6773.12749 . 28804904 PMC6051986

[37] Chieh-Yu L, Yunh-Tai H, Yi-Li C, Yi-Ju C, Wen-Shun W, Jih-Shin L, et al Incorporating Development Stratification of Taiwan Townships into Sampling Design of Large Scale Health Interview Survey. Journal of Health Management. 2006;4(1):1–22.

[38] Deyo RA, Cherkin DC, Ciol MA. Adapting a clinical comorbidity index for use with ICD-9-CM administrative databases. Journal of clinical epidemiology. 1992;45(6):613–9. 10.1016/0895-4356(92)90133-8 1607900

[39] Ministry of Health and Welfare. Taiwan Triage and Acuity Scale. 2015.

[40] Liu SW, Obermeyer Z, Chang Y, Shankar KN. Frequency of ED revisits and death among older adults after a fall. The American journal of emergency medicine. 2015;33(8):1012–8. Epub 2015/05/20. 10.1016/j.ajem.2015.04.023 25983268 PMC4962693

[41] Wang HY, Chew G, Kung CT, Chung KJ, Lee WH. The use of Charlson comorbidity index for patients revisiting the emergency department within 72 hours. Chang Gung medical journal. 2007;30(5):437–44. Epub 2007/12/08. . 18062175

[42] Foran A, Wuerth-Sarvis B, Milne WK. Bounce-back visits in a rural emergency department. Canadian journal of rural medicine: the official journal of the Society of Rural Physicians of Canada = Journal canadien de la medecine rurale: le journal officiel de la Societe de medecine rurale du Canada. 2010;15(3):108–12. Epub 2010/07/08. . 20604996

[43] Micic D, Gaetano JN, Rubin JN, Cohen RD, Sakuraba A, Rubin DT, et al Factors associated with readmission to the hospital within 30 days in patients with inflammatory bowel disease. PLoS ONE. 2017;12(8):e0182900 10.1371/journal.pone.0182900 PMC5570509. 28837634 PMC5570509

[44] Jung YW, Pak H, Lee I, Kim EH. The Effect of Diagnosis-Related Group Payment System on Quality of Care in the Field of Obstetrics and Gynecology among Korean Tertiary Hospitals. Yonsei medical journal. 2018;59(4):539–45. Epub 2018/05/12. 10.3349/ymj.2018.59.4.539 29749137 PMC5949296

[45] Vuagnat A, Yilmaz E, Roussot A, Rodwin V, Gadreau M, Bernard A, et al Did case-based payment influence surgical readmission rates in France? A retrospective study. BMJ open. 2018;8(2):e018164 Epub 2018/02/03. 10.1136/bmjopen-2017-018164 29391376 PMC5829593

[46] Knuttel FM, van der Velden BH, Loo CE, Elias SG, Wesseling J, van den Bosch MA, et al Prediction Model For Extensive Ductal Carcinoma In Situ Around Early-Stage Invasive Breast Cancer. Investigative radiology. 2016;51(7):462–8. Epub 2016/02/11. 10.1097/RLI.0000000000000255 . 26863579

[47] Intapad S, Ojeda NB, Dasinger JH, Alexander BT. Sex Differences in the Developmental Origins of Cardiovascular Disease. Physiology. 2014;29(2):122–32. 10.1152/physiol.00045.2013 PMC3949204. 24583768 PMC3949204

[48] Mueller SK, Zheng J, Orav EJ, Schnipper JL. Rates, Predictors and Variability of Interhospital Transfers: A National Evaluation. Journal of hospital medicine. 2017;12(6):435–42. Epub 2017/06/03. 10.12788/jhm.2747 . 28574533 PMC11096839

